# Global Burden of Allergies: Mechanisms of Development, Challenges in Diagnosis, and Treatment

**DOI:** 10.3390/life15060878

**Published:** 2025-05-29

**Authors:** Ewa Alska, Agata Doligalska, Katarzyna Napiórkowska-Baran, Marcin Dolina, Karolina Osińska, Anastazja Pilichowicz, Aleksandra Wojtkiewicz, Justyna Julia Kaczor, Bartłomiej Szymczak, Zbigniew Bartuzi

**Affiliations:** 1 Department of Allergology, Clinical Immunology and Internal Diseases, Collegium Medicum Bydgoszcz, Nicolaus Copernicus University Torun, 85-067 Bydgoszcz, Poland; ewa.alska@cm.umk.pl (E.A.); zbartuzi@cm.umk.pl (Z.B.); 2 Student Research Club of Clinical Immunology, Department of Allergology, Clinical Immunology and Internal Diseases, Collegium Medicum Bydgoszcz, Nicolaus Copernicus University Torun, 85-067 Bydgoszcz, Poland; doli285@wp.pl (A.D.); melminio@wp.pl (M.D.); osinska.karolinax@gmail.com (K.O.); ana-pilichowicz@wp.pl (A.P.); wojtkiewicz.ol@gmail.com (A.W.); justyna.kaczor.98@gmail.com (J.J.K.); 3 Department of Internal Medicine, Jan Biziel University Hospital No. 2 in Bydgoszcz, 85-168 Bydgoszcz, Poland; bartlomiej.szymczak1@gmail.com

**Keywords:** allergy, hypersensitivity, primary immunodeficiencies, allergens

## Abstract

Allergic diseases represent a major and growing global health concern, with increasing prevalence among both children and adults. This manuscript presents an extensive review of allergy mechanisms, epidemiology, diagnostics, and clinical challenges, highlighting the complex interplay between immune system dysregulation and environmental exposures. The authors provide a structured analysis of hypersensitivity types, with particular focus on IgE-mediated responses, and emphasize the role of immune barrier defects, epigenetics, and the microbiota in allergic pathogenesis. This manuscript explores diagnostic limitations, including test sensitivity, specificity, and the presence of hidden allergens, as well as challenges in identifying food-related or atypical allergic reactions. A novel and valuable aspect is the discussion of allergy as a potential clinical manifestation of primary immunodeficiencies, such as selective IgA deficiency, Wiskott–Aldrich syndrome, hyper-IgE syndrome, and Netherton syndrome. This review also outlines challenges in treatment, especially among polysensitized patients, and examines the psychosocial burden and complications of allergic diseases, including mental health, nutritional deficiencies, and impaired sleep. This comprehensive synthesis underscores the need for early diagnosis, multidisciplinary management, and personalized therapeutic strategies to improve quality of life of allergic patients.

## 1. Introduction

**Figure 1:**
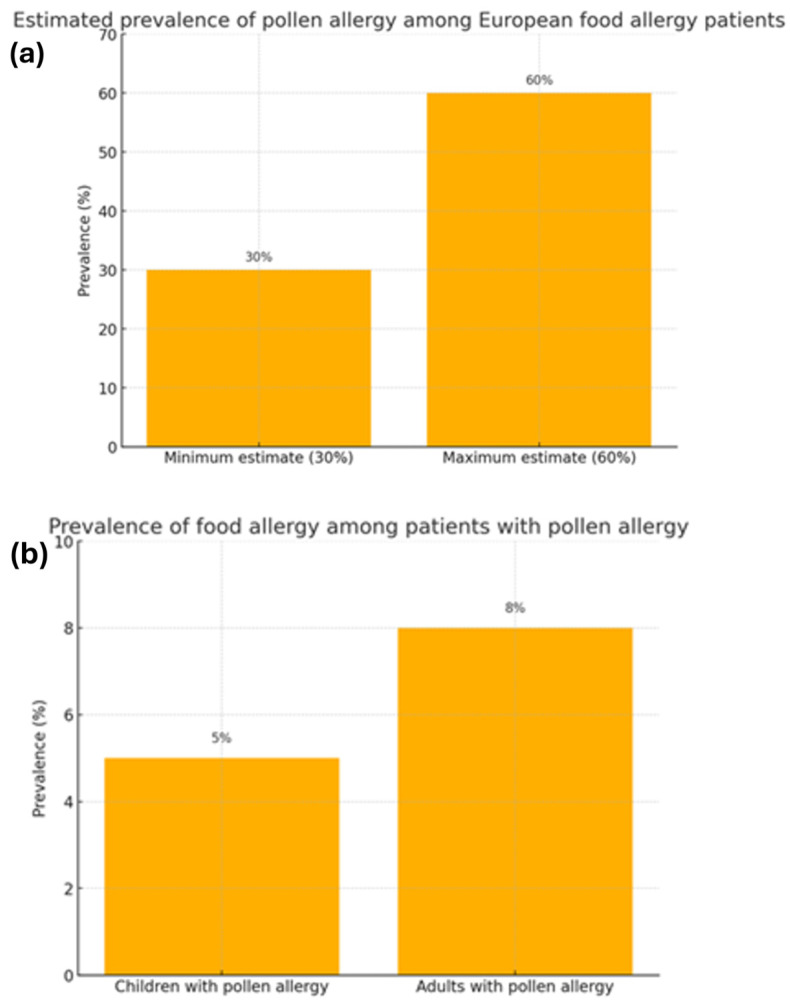
The correlation between the prevalence of food allergies and pollen allergies [2]. (**a**) Estimated prevalence of pollen allergy among European food allergy patients. (**b**) Prevalence of food allergy among patients with pollen allergy.

**Figure 2:**
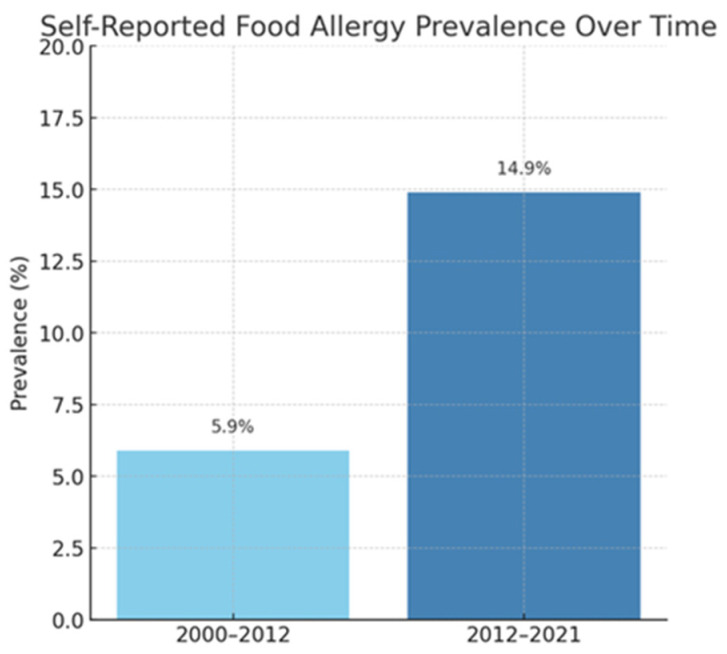
Self-reported food allergy prevalence over time [3].

Allergies are a growing health challenge affecting people of all ages. They represent a significant healthcare problem due to their prevalence. In recent years, the number of allergy cases has steadily increased due to several factors, such as environmental pollution and changes in diet and lifestyle. Climate change may significantly impact the development of respiratory allergies and asthma. Consequently, it poses a potential threat to global health by affecting the quality of food, water, and air [1]. The prevalence of allergies is widespread among both children and adults, affecting approximately 20–30% of the population (Figure 1a,b) [2]. According to the recently published literature, food allergies persist throughout life, with a reported prevalence of 19.9% in Europe (Figure 2) [3]. In globally defined statistics, the prevalence varies across different regions of the world; however, when considered a global health issue, it is estimated to affect approximately 8% of children and 10% of adults. In developed countries and urban areas, an increasing trend in allergy prevalence can be observed, likely attributed to the impact of environmental pollution [4]. Allergy symptoms can range in severity from mild skin reactions and seasonal rhinitis to severe cases of anaphylaxis, which can be life-threatening. Correct diagnosis is a key step in successfully treating allergies, but this is not always straightforward. Despite characteristic clinical signs and technological advances in diagnostic methods, determining the cause and mechanism of a given allergic reaction is often impossible. This is the reason for meticulous monitoring of allergy prevalence and its symptoms’ evolution. Over the years, symptoms have evolved in different age groups exposed to risk factors. An increased prevalence of respiratory symptoms caused by pollen allergy can also be observed. The presence of serum IgE antibodies specific to grass pollen allergens can be detected in 10–35% of young European adults [1]. A significant problem is the variety of allergens that can trigger an excessive immune system response. These can include dust mites, pollen, animal dander, food ingredients, preservatives, drugs, or chemicals in cleaning and cosmetic products. In addition, allergies are often cross-reactive—a person allergic to one allergen may also react to other allergens with a similar structure. Physicians are increasingly reporting cases of pollen-food allergy syndrome (PFAS). Cross-reactivity between pollen antigens and food allergens in sensitized individuals causes this syndrome. The variety of pollen antigens makes this problem common across all regions [2,5]. Allergy symptoms are sometimes non-specific and easily confused with other conditions, such as viral infections or food intolerances. Therefore, an accurate diagnosis is a time-consuming process that involves a variety of methods, including skin tests, molecular tests, or provocation tests [6]. In addition to the fact that diagnosis usually takes a long time, it is also limited by the low sensitivity and specificity of the tests [7]. An individualized approach to the patient and a thorough medical history tracing the entire disease history are important. When analyzing the data provided by the patient, attention should be paid to comorbidities that may aggravate or mask the first symptoms of an allergic reaction. Despite the difficulties, advances in medical science and the spread of knowledge about allergies through health education facilitate medical interventions. Appropriate therapeutic methods can significantly improve the quality of life of patients struggling with this problem.

## 2. Allergy and Hypersensitivity

The extensive terminology of allergic diseases requires the use of classifications. Although the underlying cause may be similar, the conditions usually have different mechanisms and courses of action and, therefore, require different diagnostic measures and treatments. It is worth noting that, nowadays, the terms “allergy” and “hypersensitivity” are not used interchangeably, and their meaning has been revised. “Allergy” is described as “a hypersensitivity reaction initiated by proven or strongly suspected immunologic mechanisms”. “Hypersensitivity” is defined as “conditions clinically resembling allergy that cause objectively reproducible symptoms or signs, initiated by exposure to a defined stimulus at a dose tolerated by normal subjects”. Consequently, hypersensitivity does not fulfill the criteria for allergy. In order to diagnose an allergy, the precise background of the disease must be determined, and the underlying disease must be proven by diagnostic procedures [8,9].

There are specific types of hypersensitivity. Type 1 is the immediate, IgE-dependent response. The type I response consists of two phases: a sensitization phase and an effector phase. The sensitization phase depends on T2 cell signals that regulate the production of allergen-specific immunoglobulin E (sIgE). In contrast, in the effector phase, IgE-coated immune cells form cross-links after subsequent exposure to the allergen, causing cell degranulation. Mediators, cytokines, and chemokines, including histamine, are released during this process. Inflammation and the characteristic symptoms of an allergic reaction are induced. This type of hypersensitivity may occur in patients with allergic rhinoconjunctivitis, asthma, Alzheimer’s disease, acute urticaria/angioedema, food, venom, and drug allergies [10]. Most people believe that they are caused by IgE-dependent reactions only, forgetting several other mechanisms. Allergic hypersensitivity reactions include type II cytotoxic reactions involving IgM and IgG immunoglobulins, the complement system, NK cells, and FcγR receptor phagocytes. Type II reactions are usually caused by drugs that bind to proteins on the cell membrane. The resulting complexes and anti-drug antibodies activate the complement system or are bound by the FcγR receptor on effector cells, resulting in cytolysis. Autoimmune diseases with a type II hypersensitivity reaction in their pathogenesis include immune thrombocytopenia, autoimmune hemolytic anemia (AIHA), autoimmune neutropenia, Biermer’s disease, Goodpasture’s syndrome, fetal and neonatal hemolytic disease (fetal erythroblastosis), myasthenia gravis, pemphigus, and transfusion reactions involving incompatible blood groups. Between 3 and 10 h after exposure to the allergen, a type III hypersensitivity reaction may develop in which antibodies, usually of the IgG class, bind soluble extrinsic or intrinsic antigen. This results in the formation of complexes that trigger the classical pathway of the complement system, which recruits tissue-damaging inflammatory cells. Diseases associated with type III hypersensitivity include acute hypersensitivity, pneumonia, drug-induced vasculitis, septicaemia, Arthus reaction, and autoimmune diseases such as systemic lupus erythematosus (SLA) and rheumatoid arthritis (RA) [10]. There is also a type IV-cell type, which, according to the 2023 EAACI classification, is divided into subtypes IVa, IVb, IVc, involving Th1, Th2, and Th17 lymphocytes, respectively. Exposure to exogenous or endogenous antigen triggers a local inflammatory response that attracts macrophages and monocytes. These cells engulf the antigens and present them to T lymphocytes, leading to their activation and the secretion of their respective cytokines and chemokines. Diseases resulting from type IV hypersensitivity reactions are contact dermatitis and drug hypersensitivity [11]. The epithelial barrier defect activates type V hypersensitivity reactions, producing alarmins such as IL-33, IL-25, and TSLP (thymic stromal lymphopoietin). The stated risk factors stimulate an immune response involving Th2 lymphocytes. Also, obesity can induce immune dysregulation (type VI hypersensitivity response). This occurs through increased concentrations of pro-inflammatory cytokines, neutrophils, and eosinophils in the peripheral blood and increased concentrations of acute phase factors, oxygen free radicals, and chemokines associated with increased BMI. Type VII hypersensitivity reaction, the last one, is the so-called direct cellular and inflammatory response to chemicals. At the basis of this reaction is the inhibition of cyclooxygenase 1 (COX-1) and the release of arachidonic acid-derived substances—eicosanoids. This results in reduced synthesis of prostaglandins and overproduction of cysteinyl leukotrienes (LTC4, LT4, LTE4), potent mediators of inflammation [10]. Table 1 presents the classification and mechanisms of hypersensitivity reactions.

## 3. Etiopathogenesis

Allergy is an abnormal, increased response to external stimuli, which includes different types of hypersensitivity reactions. A maladaptive type 1 immune response causes IgE-dependent allergic diseases. Contact of the allergen with epithelial barriers provokes interaction of antigen-presenting dendritic cells with naive CD4 lymphocytes. This leads to the differentiation of Th2 cells secreting IL-4 and IL-13. These lymphocytes interact with B cells, stimulating them to produce specific IgE class antibodies [12]. Combining an allergen-specific IgE class antibody (sIgE) with the FcεRI receptor on the surface of mast cells and basophils leads to the formation of cross-links and their subsequent activation. This causes cell degranulation, resulting in the release of inflammatory mediators. The role of histamine is highlighted, which causes vasodilation, promoting increased vascular blood flow and permeability. Among the molecules released is also TNF-α, which stimulates the expression of adhesion molecules on endothelial cells and increases the influx of inflammatory cells into the tissue. There are two types of reactions: immediate and late. The former is caused by the activation of mast cells and the action of histamine, prostaglandins, and other rapidly produced mediators. In contrast, the induced synthesis of chemokines, leukotrienes, and cytokines causes the late phase. This leads to the recruitment of Th2 lymphocytes and eosinophils to the site of infection [13].

Continued exposure to the allergen provokes a chronic inflammatory process, leading to permanent tissue damage and remodeling and, consequently, fibrosis and parenchymal loss. Among the factors implicated in the development of allergy are epigenetic changes in nuclear and mitochondrial DNA. Studies report that the cause of asthma in women may be due to dysfunction of the mitochondrial genes MT-ND2 and MT-RNR2. In contrast, in men, a mutation in the mitochondrial cytochrome b gene contributes to the development of the disease [14]. It has been noted that reduced methylation of 21 CpG regions is associated with the onset of allergy in childhood from 4 to 16 years of age. These methylation sites are common to asthma, rhinitis, and eczema and are associated with higher eosinophil, CD8 memory lymphocyte, and NK cell activity [14,15]. An imbalance between histone acetyltransferase (HAT) and histone deacetylase (HDAC) activity, which regulates the level of gene expression, is also seen in patients with asthma. Increased HDAC activity in allergic rhinitis results in increased pro-inflammatory cytokines and decreased anti-inflammatory cytokines and leads to nasal epithelial dysfunction. Blocking HDACs promotes the secretion of IL-10 (an anti-inflammatory cytokine) and prevents excessive activation of immune cells. Microbial colonization begins at birth, and the composition of the microbiota plays an important role in immune function. The types of birth process, feeding, and the use of antibiotics in early life affect the composition of the gut microbiota. Antibiotic use during pregnancy and the postnatal period increases the risk of allergic diseases in infants. The microbiota of children exposed to antibiotics shows reduced number of Bacteroidetes and Bifidobacterium bacteria, which beneficially affect the immune system, and increased Proteus bacteria, which are a common etiological agent of urinary tract infections. Early exposure to antibiotics is also associated with the development of childhood asthma, allergic rhinitis, and skin atopy. Haemophilus, Moraxella, and Neisseria spp., the pathogens that cause respiratory tract infections, have also been observed among the airway microbiota of patients with asthma, while Proteus has also been found in mild as well as severe cases of asthma [14].

## 4. Structure of Allergens

The biochemical structure of allergens and their properties are important factors in inducing an immune response. The main types of allergens include proteases, lipid-binding/transfer proteins, actin-binding proteins, calcium-binding proteins, α-amylase/trypsin inhibitors, and pectin lyase. The common feature of allergens is hydrophilicity, which facilitates their dissolution in body fluids and the induction of an immune response. Allergens that trigger a response in the respiratory system must be suspended in the air, but this property depends on the climate. In the case of greater rainfall, the concentration of allergens decreases. This is due to proteins dissolving in rainwater and binding to other molecules. The cat allergen (Fel d 1) remains in the air for a long time and is adhesive, so it adheres to clothes, which makes it easier to transfer [16]. For food allergens, resistance to digestive enzymes and heat treatment is important. Egg allergens ovalbumin (Gal d 2) and ovotransferrin (Gal d 3) are heat sensitive, which is why most children with egg allergy tolerate hard-boiled or baked eggs, while consumption of raw or undercooked eggs is associated with an allergic reaction. Ovomucoid (Gal d 1) is highly thermostable, therefore heating does not contribute to reducing the allergenic properties. Only the presence of wheat causes the thermostability of ovomucoid to decrease [17]. Among the allergenic carbohydrates, cross-reactive carbohydrate determinants, alpha-gal, and galactooligosaccharides can be distinguished. The cross-reactive carbohydrate determinants include glycans. Rare cases of skin reactions to cross-reactive carbohydrate determinants in oilseed rape have been reported. Glycosylation of allergens can affect the tertiary structure of proteins and inhibit protein cleavage by digestive enzymes, thereby increasing antigenicity. Lipids also participate in the immune response. Sphingolipids contained in milk can stimulate NK cells and induce the production of IL-4, IL-5, IL-13 in patients with milk allergy [16]. Lipid transfer proteins (LTPs) are widely distributed among fruits and pollens. LTPs have a special pocket that allows them to bind to lipids. The CPT-PHS ligand present in LTP stimulates the production of proinflammatory cytokines, thereby increasing the immune response and the production of IgE antibodies [18]. Nickel is one of the most common metals that cause allergies. Prolonged and direct skin contact with objects containing nickel causes it to react with sweat. The released nickel ions are absorbed through the skin and initiate an allergic reaction [19]. Table 2 presents the relevant types of allergens presented with characteristics and examples. Research into the properties of allergens and their molecular structure and stability in the environment is important for the development of effective therapies, which will help reduce the risk of severe allergic reactions and anaphylaxis.

## 5. Difficulties Related to Allergic Diseases

The complex mechanism of allergic reactions and the range of symptoms, that can imitate other diseases, make diagnosing allergic diseases extremely difficult. It can imitate a variety of other illnesses and the intricate processes of immunological reactions. Finding a specific allergen can be challenging, particularly when the patient is susceptible to many possible triggers. Furthermore, it can be challenging to directly correlate symptoms with a particular allergen because allergy test results are not always clear-cut, and symptoms can manifest gradually. Accurate diagnosis necessitates a comprehensive medical interview, appropriate testing, and a holistic approach to the patient. The summary of the sections is presented in Table 3.

### 5.1. Hidden Allergens

An important problem in diagnosing allergies is the presence of hidden allergens, which can cause severe reactions to seemingly unrelated products. These compounds can be contained as food additives or incorporated into compounded products, but they can also come from contaminants, such as grain mites. By identifying hidden allergens, the probability of a patient developing an allergic reaction can be reduced, and the patient’s comfort can be increased.

The legal regulations pertaining to the labelling and markings of those substances vary across different nations. Compound products contain many constituents, including preservatives, food additives and spices. Consequently, there is a possibility that the labeling of substances or the assessment of their allergenic potential during processing may be inaccurate. The European Food Safety Authority (EFSA) requires peanuts, soy, and lupin to be labeled in products. Common antigenic epitopes in legumes promote cross-reactions, which makes allergy diagnosis even more difficult [26].

A notable paradigm of the hidden allergens is celery, which is commonly found in soups, meat dishes, sauces, pizzas, condiments, and ready meals. Allergic reactions appear even at very low doses—the estimated amount likely to cause a reaction in 10% of the study population is 1.6 mg of protein [27]. Celery is one of the foods associated with food-dependent, exercise-induced anaphylaxis (so-called FDEIA). Its allergens also cross-react with other allergens, such as birch pollen and mugwort. The primary celery allergen, Api g1, is thermostable, so symptoms also occur after eating the cooked product and the spices it contains. Another widely used product is mustard, which enhances the flavor of a given dish. It is also used as an additive in prepared meals, so there is a risk of it being overlooked as an allergen. Mustard has a high allergenic potential—a dose of 0.05 mg of the primary allergen causes symptoms [28]. The complexity of identifying a particular allergen is particularly pronounced in spices. Fenugreek, an ingredient in curry, has been identified as a cause of reactions to spicy foods and Indian cuisine. An allergy to it may co-occur with a peanut allergy or occur independently. For this reason, the physical examination plays an important role in which the patient should be questioned in detail about the products consumed and their additives.

Legumes represent a significant cause of allergies. Among these, peanuts and soybeans have been identified as the most prevalent. Lupin is also on the rise and is increasingly being added to gluten-free flour or high-protein foods. Lupin allergens are thermostable and cross-react with peanut allergens. The European Food Safety Authority (EFSA) stipulates that labeling is mandatory for products containing lupin, akin to the labeling requirements for peanuts and soy. Legumes share common antigenic epitopes, making cross-reactivity between species common. Natural food colors with allergenic potential include carmine, which is used to color food as well as cosmetic products. Sulfites contained in white and pink wine, light fruit juices, dried fruit and vegetables, and salted cod are also important. It has been shown that people with asthma are more likely to react to sulfites. Non-food products may also contain hidden food allergens. For example, buckwheat hulls used in pillows can cause asthma and allergic rhinitis [29]. A case of anaphylactic reaction to chlorhexidine, a common antiseptic, has also been reported [30]. Natural food dyes with allergenic potential include carmine, which is used to color food and cosmetic products. A case of a 32-year-old woman with bullous eruptions, redness, and itching around the eyelids caused by pink eye shadows with the addition of carmine has been described. The same patient had an anaphylactic reaction to fried pink fish sausages containing the aforementioned ingredient [20].

Allergy to the parasite Anisakis simplex, which infects fish such as cod and salmon, can occur when eating raw or marinated fish. We can divide the allergens into somatic allergens (derived from dead or live larvae) and excretory antigens, which are released when the larvae are expelled from the host’s gastrointestinal tract or surgically removed [31]. Some of the symptoms associated with allergy may only occur when so-called ‘triggers’ are present, which include ambient temperature, exercise, alcohol, and non-steroidal anti-inflammatory drugs (NSAIDs). According to studies, exercise lowers the threshold, increases the severity of the reaction to food (especially wheat), and is associated with exercise-induced anaphylactic shock (FDEIA). Among drugs, aspirin is the most common trigger for reactions. When admitting such a patient, a thorough physical examination ought to be carried out, including the circumstances of the symptoms, the food consumed, and the presence of additional factors. Most patients report episodes starting with pruritus, redness, and urticaria during exercise. Food is usually consumed 2 to 3 h before planned exercise. In some cases, symptoms may also occur regardless of physical activity. These are when the allergenic food is consumed in large quantities or if concomitant factors such as alcohol consumption are present [32].

### 5.2. Atypical Symptoms

Kounis syndrome is a rare disorder characterized by an allergic reaction that leads to acute coronary symptoms [21]. Its etiology is most often attributed to the administration of antibiotics, non-steroidal anti-inflammatory drugs, and Hymenoptera venom [33]. The main allergenic proteins in the venom of hymenopteran insects from the Vespoidea superfamily, such as wasps (*Vespula* spp., *Polistes dominula*) and hornets (*Vespa crabro*), are antigen 5 proteins (Ag 5). Despite their yet unknown biological function, the proteins exhibit high immunogenicity, thereby positioning them among the most salient allergens in the diagnostic assessment of venom allergies. Due to their significant cross-reactivity, Ag 5 proteins complicate the precise identification of the primary sensitizing venom in patients allergic to both bee and wasp venom [34].

Kounis syndrome involves the activation of mast cells by contact of the allergen with specific IgE antibodies and by activation of the complement system. This releases inflammatory mediators such as histamine, cytokines, leukotrienes, and proteases. These mediators cause coronary vasoconstriction, platelet activation, and destabilization of atherosclerotic plaques, resulting in myocardial ischemia [21,33,35,36]. The occurrence of Kounis syndrome may be associated with the use of a nonsteroidal anti-inflammatory drug, diclofenac, which is a derivative of phenylacetic acid. All NSAIDs inhibit the activity of cyclooxygenase-1 (COX-1), which shifts the metabolism of arachidonic acid toward the lipoxygenase pathway. This results in increased release of inflammatory mediators, such as histamine and tryptase, from mast cells and eosinophils [21].

An allergic reaction may cause acute pancreatitis. Consumption of banana, kiwi, milk, tuna, mackerel, and mustard can cause swelling of the Vater’s ampulla, which leads to reflux of bile and pancreatic enzymes, which causes acute pancreatitis. Few such cases have been described, yet their common element was the disappearance of symptoms after cessation of food consumption, causing the allergic reaction [37,38,39,40]. A concise characteristics of selected allergens causing specific symptoms of allergic reactions is shown in Table 4. In bananas, six proteins responsible for triggering allergic reactions have been identified: Mus a 1 (profilin), Mus a 2 (class 1 chitinase), Mus a 3 (non-specific lipid transfer protein), Mus a 4 (thaumatin-like protein), Mus a 5 (β-1,3-glucanase), and Mus a 6 (ascorbate peroxidase). The primary allergen is Mus a 1 (profilin), a member of the actin-binding protein family, which may cause cross-reactivity with plant pollens [41,42]. In kiwi, Act d 1 is the key allergen, which is a protein derived from the PR-10 family, homologous to Bet v 1 from birch pollen, along with Act d 9 and the aforementioned profilin, which are also responsible for cross-reactions with pollens and may lead to pollen-food allergy syndrome [43,44]. Bovine milk contains β-lactoglobulin (Bos d 5), a lipocalin family protein, which is the main whey allergen and may induce strong allergic reactions [45]. In fish, such as tuna and mackerel, the leading allergen is parvalbumin, a member of the calcium-binding protein family, which is resistant to high temperature and digestion, which increases its allergenicity [46,47]. Mustard contains Sin a 1, a 2S-albumin family protein, the fundamental allergen in mustard seeds [47,48]. In most cases, allergic reactions have particular symptoms, and their diagnosis is not problematic. Unfortunately, it is sometimes difficult to link the symptoms to a specific allergen or the allergic reaction itself [21,23].

### 5.3. Allergy as a Mask of Primary Immunodeficiency

Primary immunodeficiencies (PIDs) are currently known as inborn errors of immunity (IEIs)—this is a more appropriate name as it suggests general abnormalities in the functioning of the immune system and not only increased susceptibility to infections [49]. In individuals with IEIs, even though the most prevalent symptom is the mentioned elevated risk of infection, which usually manifests in a more rapid and severe clinical course, these individuals also demonstrate an increased vulnerability to autoimmune diseases, including allergies. The pathogenesis of allergies and IEIs is based on the improper functioning of the immune system [50,51]. In allergies, the immune system’s response to allergens involves the production of specific antibodies, which, after binding to the antigen, stimulate mast cells to release the contents of their follicles, and clinically manifests as itching, sneezing, runny nose, bronchospasm, and hives. Allergy is the production of unnecessary antibodies, which cause the immune system to react to an antigen that does not threaten the body [50,52]. Inborn errors of immunity result from the absence, weakened, or improper functioning of the immune system components, which leads to impaired defense reactions of the body [49,50,51]. Due to the increased popularity of allergies, inborn immunodeficiencies are often omitted in diagnostics or left as a last resort, and their incidence is relatively high [49,51]. Many of these conditions can manifest with symptoms resembling allergy, which is a diagnostic challenge and can delay diagnosis [50,51].

Primary immunodeficiencies are relatively rare, occurring about once in ten thousand live births. However, selective IgA deficiency occurs much more often, especially in isolated populations or populations with high inbreeding [53,54]. In selective IgA deficiency, an increased tendency to allergic reactions is observed due to the fact that immunoglobulin A plays a key role in protecting the mucous membranes against pathogens, and its deficiency promotes the penetration of allergens and the development of allergic reactions, such as asthma, allergic rhinitis, or atopic dermatitis. Patients with this deficiency also have recurrent upper and lower respiratory tract infections, which can lead to a false diagnosis of chronic allergy [24,53,55,56].

Wiskott–Aldrich syndrome (WAS) is a recessive, sex-linked hereditary disease occurring only in males. It is caused by a mutation of the WAS gene, which encodes a protein regulating the function of blood progenitor cells, thrombocytes, lymphocytes, and macrophages. It manifests itself with a triad of disorders: recurrent infections, severe skin lesions, and bleeding diathesis, resulting from thrombocytopenia [57,58]. Additionally, in its course, an increased frequency of IgE-dependent food allergies, autoimmune diseases, and cancers is observed [58,59]. Elevated IgE levels are also observed in these patients, complicating the distinction of the disease from classic allergic diseases [54,55].

Job’s syndrome, also known as hyper-IgE syndrome, is an uncommon disease with a genetic basis leading to immune disorders. It is characterized by high levels of immunoglobulin E, frequent infections, and skin lesions [60,61,62,63,64]. There are two main forms of the disease: autosomal dominant, resulting from mutations in the STAT3 gene, and autosomal recessive, associated with mutations in the DOCK8 or PGM3 genes [60,61,62,64]. The basic feature of the disease is a significantly elevated level of IgE, which can reach values of several thousand IU/mL. Skin symptoms, such as eczema, and a tendency to allergic reactions may resemble IgE-dependent allergy, which complicates the diagnostic process [62,64]. However, the key difference with allergies is the weakened function of the immune system—patients have impaired activity of Th17 lymphocytes, which weakens the body’s ability to fight infections and predisposes to severe infections, which is the reason that Job’s syndrome often remains undiagnosed and is confused with allergy [25,54,60,61,62,63,64].

Netherton syndrome is a rare hereditary skin disease associated with weakened immunity. It is transmitted in an autosomal recessive manner [65,66]. It is caused by a mutation in the SPINK5 gene located on chromosome 5 [66,67]. This gene is responsible for the production of the LEKTI protein, which regulates the activity of certain enzymes in the skin and mucous membranes. If LEKTI does not function properly, the skin and gastrointestinal tract protective barrier becomes permeable, resulting in easier penetration of allergens into the body and promoting the development of food allergies and other atopic diseases [65,66,67]. Patients also have a weakened immune system, which is manifested, among others, by a reduced ability of the body to produce antibodies and improper functioning of NK cells, which play a key role in cellular immunity. The main symptoms include characteristic skin lesions—red, flaky skin and very brittle, fragile hair. Children with this syndrome often suffer from allergies, including bronchial asthma, and are prone to recurrent infections and digestive problems, such as diarrhea. Allergic symptoms may dominate the clinical picture, so Netherton’s syndrome is sometimes confused with classic allergic diseases [54,66,67]. As a result, this may lead to delayed diagnosis and inappropriate treatment [65,66,67].

Diagnosis of PIDs requires a detailed interview, a thorough assessment of the patient’s medical history, a determination of the family history of immune disorders, and immunological tests. Awareness of the co-occurrence of allergies and primary immunodeficiencies is important. The process of diagnosis is often complicated due to the fact that allergic symptoms are typically more severe and may mask the primary symptoms of IEIs, which include elevated susceptibility and an exacerbated course of infections. Early detection of inborn errors of immunity in patients with allergic symptoms is crucial for implementing appropriate treatment and preventing serious health complications [49,50,51,52,54,68].

### 5.4. The Diagnosis of Allergic Diseases Is Characterized by Numerous Limitations

The diagnostic process for allergic diseases is complex and multifactorial. Despite significant advances in laboratory techniques, no universal test can confirm or rule out allergy with high specificity across diverse clinical scenarios. The subsequent sections are a summary of general diagnostic limitations and an overview of specific challenges associated with common allergic disorders.

#### 5.4.1. Limitations of Standard Allergy Testing

The measurement of serum-specific IgE (sIgE) is a commonly used diagnostic tool; however, its clinical utility is limited by modest sensitivity and specificity. Numerous studies have shown that positive sIgE or skin prick test (SPT) results do not necessarily reflect a clinically relevant allergy. In contrast, negative results, particularly in the presence of strong clinical suspicion, do not reliably exclude the diagnosis. This is especially relevant in non-IgE-mediated hypersensitivity reactions [69,70].

The diagnostic value of IgE-based tests is also affected by the biological properties of IgE: the half-life of free circulating IgE is relatively short (approximately 2–3 days), and a significant proportion (nearly 50%) resides in extravascular compartments, further complicating the interpretation of serum levels [71].

The double-blind placebo-controlled food challenge (DBPCFC) remains the gold standard for diagnosing food allergies. However, its application is limited by cost, time requirements, the need for specialized centers, and the risk of inducing serious adverse reactions, including anaphylaxis. Despite its status, studies indicate that DBPCFC confirms only 30–40% of allergy cases suspected based on clinical history alone [72,73].

The Basophil Activation Test (BAT) is an emerging tool with significant potential. This functional ex vivo assay measures basophil degranulation upon allergen stimulation, typically using flow cytometry and markers such as CD63 and CD203c. Although BAT has shown high predictive value, it remains underutilized due to a lack of standardization and validation protocols between laboratories [74].

Additionally, elevated IgE levels may be observed in numerous non-allergic conditions, including hematologic malignancies (e.g., IgE myeloma, Hodgkin lymphoma), immunodeficiencies (e.g., Wiskott–Aldrich syndrome, Job’s syndrome), parasitic infections, autoimmune diseases, and certain viral or bacterial infections [75].

#### 5.4.2. Challenges in Allergic Diagnostics

##### Asthma

Asthma diagnosis is complicated by the absence of a single definitive test and the variability of clinical manifestations. Symptoms such as wheezing, shortness of breath, cough, and chest tightness are nonspecific and fluctuate over time, contributing to underdiagnosis and overdiagnosis [76,77]. Spirometry with bronchodilator reversibility remains the primary diagnostic method, but expected results do not exclude the disease [78]. Emerging biomarkers such as fractional exhaled nitric oxide (FeNO) and peripheral blood eosinophil counts assist in identifying eosinophilic phenotypes, although they remain underused in clinical practice [76]. Current GINA guidelines advocate for phenotype- and endotype-driven diagnostic approaches [79].

##### Allergic Rhinitis (AR)

AR diagnosis relies primarily on clinical history and confirmatory testing using SPT or sIgE. However, the correlation between test results and symptoms is often poor. Some patients exhibit rhinitis-like symptoms due to non-IgE-mediated mechanisms or mucosal hyperreactivity. In uncertain cases, nasal provocation testing may be helpful but is rarely performed due to limited availability [80].

##### Atopic Dermatitis (AD)

There is no definitive laboratory test for diagnosing AD. Clinical diagnosis is typically based on the Hanifin and Rajka criteria. A major challenge lies in differentiating AD from allergic contact dermatitis (ACD), which often coexists. In such cases, patch testing is essential but remains underutilized [81].

##### Drug Allergy

Drug hypersensitivity reactions present a significant diagnostic challenge due to the diversity of underlying immunologic mechanisms. Skin testing and in vitro assays may aid in diagnosis; however, the drug provocation test (DPT) remains the diagnostic gold standard. DPT, however, carries inherent risks and must be conducted under strict medical supervision [82,83].

##### Insect Venom Allergy

Diagnosis is typically based on SPT, sIgE testing, and, in some cases, sting provocation. Diagnostic accuracy is reduced due to cross-reactivity between venoms and the frequent presence of underlying mast cell disorders such as systemic mastocytosis [84,85].

##### Allergic Contact Dermatitis (ACD)

ACD is diagnosed primarily through patch testing, which may present interpretation difficulties, especially when atopic dermatitis coexists. Environmental and occupational exposures are often overlooked, leading to missed or delayed diagnoses [86].

### 5.5. Difficulties Associated with the Treatment of Allergic Diseases

#### 5.5.1. Polysensitization—Sensitization to Multiple Allergens

Treatment of polysensitized patients remains a challenge for allergists. There are no general recommendations for the practical approach to these patients, therefore, clinical management differs between countries. In the USA, most AIT (allergy immunotherapy) prescriptions include mixes of multiple allergens, while in Europe mixtures of allergen extracts account for only 20–40% of prescriptions. The Allermix survey, conducted in 2016 in 19 countries, showed that there are many differences in treating polysensitized patients worldwide—according to information reported by 1029 clinicians, 58% of polysensitized patients received AIT with single allergen and 48% received AIT with > 1 allergen extracts including mixed AIT (24%) or simultaneous multiple AIT (18%) [87]. Even though current evidence on AIT with multiple, non-cross-reactive allergens in polysensitized patients is insufficient, it is a common practice among allergists worldwide. Therefore, more supporting data derived from future studies are needed to standardize the approach to polysensitized patients [88].

#### 5.5.2. Food Allergies

One of the difficulties in treating allergies is the fact that managing food allergies changes over the course of life. Efficacy and safety of therapies may differ by age group of patients. For example, epicutaneous immunotherapy with a commercial product turned out to be ineffective in older children. Studies suggest that oral immunotherapy and sublingual immunotherapy seem to be more effective in very young children than in other patients. Adherence to elimination diets vary in different age groups. Transition of responsibilities from parents to children should be a process. Increasing independence of teenagers means more risk-taking behaviors which is associated with ingesting potentially risky foods. Management of food allergy among college-aged individuals seems to be particularly poor [89]. Further studies are needed to identify methods of treatment adjusted to patients of different ages in order to provide optimized care.

#### 5.5.3. Asthma

The treatment of allergic asthma also presents challenges related to the patient’s age. A critical moment in the course of allergic asthma management is the transition from pediatric care to adult care. Many children reach remission during puberty and that is why they rarely attend follow-ups and do not transition to adult healthcare until they experience acute asthma attacks [90]. Consequently, they have poor adherence to therapy and asthma control.

#### 5.5.4. Pruritus

Pruritus is a key symptom in allergic diseases [91]. Though treatment of pruritus remains challenging and unsatisfactory in general. Single-center, cross-sectional, investigator-driven study conducted in a tertiary referral center among 126 patients suffering from pruritus in the course of various skin diseases (with atopic dermatitis being the most common disease) showed that patients often consider conventional pruritus therapies insufficient. The subjective efficacy of oral antihistamines, cortisone tablets, and all topicals was rated only intermediately helpful [92].

#### 5.5.5. Biological Treatments

Although the development of biological treatments offers a broad spectrum of new and efficient treatment options for allergies, these therapies are still cost-intensive [93]. Access to biologic drugs is usually limited because of high prices. Therefore, before initiating biological treatment, clinicians should assess if the improvement in patient health will be sufficient to justify the expense and if other, less costly treatments have been optimized before considering a biologic [94].

#### 5.5.6. Allergen Immunotherapy

Allergen immunotherapy offers a more permanent solution than drugs, which only treat symptoms and not the cause, but on the other hand, it requires accurate prescription and monitoring. It is a therapeutic approach that includes repeated administration of allergen extracts. AIT comes in several forms, each delivering allergen extracts to the body through a different route. Subcutaneous immunotherapy and sublingual immunotherapy can be offered to patients with allergies to inhalant allergens. Subcutaneous immunotherapy is used among individuals at risk of anaphylaxis from venoms. Oral immunotherapy for food allergies is still an experimental approach, although recently an oral peanut product was approved for use in patients with peanut allergy. Compared with inhalant allergen immunotherapy, oral peanut immunotherapy has not induced long-term tolerance, even at high doses. Moreover, it causes gastrointestinal side effects. Although subcutaneous immunotherapy is considered the gold standard, it is associated with the risk of allergic side effects, even anaphylaxis, and that is why it necessitates specialist supervision. Currently, novel immunotherapy strategies are being developed to improve AIT by reducing side effects, increasing efficacy, and achieving more durable long-term tolerance with shorter, more convenient courses. These include recombinant allergens and hypoallergenic variants for immunotherapy, which match the patient’s sensitivities and may reduce the risk of IgE sensitization to irrelevant allergens. Other innovative strategies include DNA-based vaccines that have been tested in mouse models of allergy. However, there are concerns about their use in humans due to the theoretical risk of incorporation of plasmid DNA into the human genome, causing carcinogenesis. Current approaches may be further improved in the future, as well as through the potential for a combination of allergens with immune modifiers or monoclonal antibodies that target the T helper 2 cell pathway. New data regarding the effects of AIT with traditional allergens by alternative routes have become available [95]. Innovative molecular approaches provide new opportunities to improve conventional immunotherapy in the future.

### 5.6. The Financial Challenges of Allergy Diagnosis and Treatment

In 2020, the U.S. Food and Drug Administration (FDA) approved a standard OIT (oral immunotherapy) product (Palforzia) for peanut allergy that retails for $890, which is still a high price despite the manufacturer’s patient assistance programs. Additional costs associated with the OIT include training medical staff in administering doses and treating allergic reactions [96]. On the other hand, the use of subcutaneous immunotherapy (SCIT) in patients with IgE-mediated respiratory diseases contributed to reducing treatment costs compared to pharmacotherapy alone. At least three years of maintenance therapy reduces the costs associated with prolonged maintenance therapy and reduces the financial burden on health facilities [97]. In a study evaluating the actual cost profiles of allergic rhinitis in the United States, after one year of AIT, the AIT cohort showed a decrease in costs in the categories assessed compared with the non-AIT cohort. Annual health care costs for patients with AIT were $11,612 ± $24,797, and for non-AIT cohorts were $7815 ± $27,041 [98]. The effectiveness of therapy has been shown to be influenced by the drug administration route and the treatment regimen’s complexity. It seems that a more convenient option for patients is SLIT, taken orally once a day. Early initiation of AIT may prevent more severe disease progression and minimize comorbidities associated with allergy. Effective therapy will positively impact the quality of life of these patients and their work efficiency, which will be economically beneficial for the economy of a given country. Educational support for healthcare professionals greatly improves patient compliance, reducing healthcare costs. A 2020 study showed that workshops improving the educational competencies of nurses significantly improved the treatment of patients with asthma, allergies, and COPD [99]. This implies that educational interventions focusing on adherence aimed at healthcare professionals can be offered regularly. More and more tests, both expensive and cheaper, allow for the diagnosis of allergies. The readily available and relatively cheap ones include skin prick, serum IgE, and provocation tests. The component-resolved diagnostics, basophil activation tests, bead-based epitope assays, molecular diagnostics, and artificial intelligence applications are more precise, expensive, and not necessarily readily available. Nevertheless, in the future a wide range of diagnostic possibilities will allow for the correct diagnosis, and integrating advanced technologies with established approaches improves patient care and compliance [100].

### 5.7. Complications of Allergic Disease

Allergic diseases affect quality of life and mental health. Several studies have shown a decline in the quality of life (QoL) in allergic diseases. A study conducted among patients with allergic diseases (446 with atopic dermatitis, 483 with asthma and 2353 with allergic rhinitis) in South Korea confirmed that the QoL is poor in allergic disease patients throughout their lives compared to non-allergic controls [101]. Moreover, it indicated that the percentages of patients who experienced significant mental health problems (strong psychological stress, psychiatric consultations, diagnosis of depression) were higher in the group of participants with allergic diseases compared to the non-allergic control group. A cross-sectional study including 596 patients with allergic symptoms or previously diagnosed allergies suggested that there may be a correlation between allergic diseases and/or urticaria with a reduced QoL and a higher depression rate [102]. Experiencing a life-threatening anaphylactic reaction to Hymenoptera venom can have a long-lasting negative impact on patients’ quality of life [103]. Food allergies have a significant impact on QoL compared to other chronic illnesses due to strict avoidance of allergenic foods, which is a primary recommendation in food allergy management. Patients devote a significant amount of time to thinking about food and its impact on their bodies. It may contribute to the development of body image disturbances. Moreover, food allergies result in increased incidence of eating disorders such as anorexia nervosa or avoidant/restrictive food intake disorder. There is some evidence that bulimia nervosa might be associated with food allergies, although it is limited and further studies are needed [104]. Research results suggest that 30% of patients receiving intensive feeding therapy also present food allergy, which indicates that feeding disorders might be more prevalent among people with food allergy than within the general population [105]. In addition to psychosocial impacts, dietary restrictions associated with food allergies may lead to physical consequences such as increased risk of specific macronutrient deficiencies, insufficient energy intake and impaired growth indices [104]. Studies have shown that food allergies are associated with reduced intake of macronutrients, especially proteins and with micronutrient deficiencies, particularly vitamin A and iron. Cow milk allergy is a risk factor for iron-deficient anemia. A large retrospective study indicated that children presenting with cow milk allergies are significantly shorter and weigh less than nonallergic children. Dietary restrictions in children with food allergy were also linked with increased disease severity [106]. According to research findings, allergic diseases can influence sleep and subsequent daytime functioning. Studies have suggested that allergic rhinitis is an important factor influencing sleep disturbance. A cross-sectional study conducted among 8645 Korean female nurses with allergic rhinitis confirmed that allergic rhinitis is significantly associated with sleep disturbances and fatigue [107]. A systematic review and meta-analysis examining the associations of allergic rhinitis with sleep duration and sleep impairment suggested that allergic rhinitis might be linked with a higher risk of nocturnal sleep-related dysfunctions (insomnia, nocturnal enuresis, restless sleep, sleep-disordered breathing, obstructive sleep apnea, snoring) and daytime sleep-related dysfunctions (difficulty waking up, daytime sleepiness, morning headache, the use of sleeping pills) [108]. A prospective, case-controlled study conducted among 73 children with allergic conjunctivitis and 81 healthy, age-matched children who served as controls revealed that allergic conjunctivitis potentially disturbs the sleep quality of pediatric patients. Further studies to examine whether anti-allergic therapy can improve the quality of sleep in patients with allergic conjunctivitis are needed [109]. The susceptibility of children presenting with asthma to sleep-breathing disorders is well-established. A cross-sectional study conducted among 98 adolescents with asthma revealed a substantial frequency of sleep-breathing disorders in this group of patients [110]. Atopic dermatitis is linked with sleep disturbances in 47–80% of children and 33–90% of adults. Due to the Federal Drug Administration’s Patient-Focused Drug Development Initiative, sleep is one of the three most bothersome symptoms among patients with atopic dermatitis [111].

## 6. Conclusions

Allergies are a health condition that affects the population worldwide, contributing to a lower quality of life in the society. A wide range of risk factors and symptoms complicates the diagnostic process, and as a result, the identification of allergic reactions remains challenging in many cases. The incidence of allergies varies depending on the geographic region, though it is recognized as a universal health concern.

The etiology of allergies is multifactorial, yet the predominant etiology is considered to be a dysfunction in the immune system.

The diagnostic process utilizes a variety of methods; however, a significant proportion of these approaches are characterized by limitations. A patient’s sensitivity to multiple allergens, the lack of specificity in allergy tests, and the potential of allergic reactions triggered by disease states represent additional challenges. It is essential to acknowledge inborn errors of immunity as one of the contributing factors to the development of allergies, in order to prevent further health complications. Currently, conventional therapies such as corticosteroids or antihistamines are ineffective, while advanced biological treatments incur substantial economic costs, thereby creating a significant financial barrier for patients and healthcare systems. Allergies not only have a profound impact on quality of life, but also contribute to the development of somatic and psychological disorders.

## Figures and Tables

**Table 1:** Types of hypersensitivity reactions—classification and mechanisms. Abbreviations: AIHA—Autoimmune Hemolytic Anemia, FPIES—Food Protein-Induced Enterocolitis Syndrome, ILC—Innate Lymphoid Cells, NSAIDs—Non-steroidal Anti-inflammatory Drugs, RA—Rheumatoid Arthritis, ROS—Reactive Oxygen Species, SLE—Systemic Lupus Erythematosus, Th—T helper cell, TSLP—Thymic Stromal Lymphopoietin [5,6,7].

Type I	Type II	Type III	Type IV	Type V	Type VI	Type VII
Immediate/Anaphylactic	Cytotoxic	Immune complex-mediated	Delayed/Cell-mediated	Epithelial barrier defect	Metabolically induced	Direct cellular and inflammatory reaction to chemicals
B cells, IgE, Th2, ILC2, IL-4, IL-13	B cells, IgG, IgM, complement system	IgM, IgG, antigen–antibody complexes	ILCs, T cells, NK cells, eosinophils, Th17	IL-33, IL-25, TSLP, Th2-type inflammation	Elevated cytokines, neutrophils, eosinophils, acute phase reactants, ROS, chemokines	Overproduction of LTC4, LTD4, LTE4, Th2/Treg imbalance
Allergic rhinitis, asthma, food allergy, insect venom allergy, drug hypersensitivity	Immune thrombocytopenia, AIHA, autoimmune neutropenia, pernicious anemia, Goodpasture’s syndrome, hemolytic disease of the newborn, myasthenia, pemphigus, transfusion reactions	Acute hypersensitivity, pneumonitis, drug-induced vasculitis, serum sickness, Arthus reaction, SLE, RA	Contact dermatitis, drug hypersensitivity	Asthma, allergic rhinitis, chronic rhinosinusitis, atopic dermatitis, FPIES, eosinophilic esophagitis, celiac disease	Obesity, asthma, histamine-related disorders	NSAID cross-reactivity, NSAID-exacerbated skin diseases, acute urticaria/angioedema triggered by NSAIDs

**Table 2:** Types of allergens with characteristics and examples [16,17,18,19].

Type of Allergen	Characteristic	Examples
Proteins	Most common, 6 groups: proteases, lipid transfer proteins, actin-binding proteins, calcium-binding proteins, α-amylase/trypsin inhibitors, pectin lyase	Fel d 1, ovomucoid (Gal d 1), ovalbumin (Gal d 2), ovotransferrin (Gal d 3),
Carbohydrates	Cross-reactive carbohydrate determinants, alpha-gal, galactooligosaccharides	Glycans in rapeseed
Lipids	Low allergenicity, stimulation of pro-inflammatory cytokine production	Sphingolipids in cow’s milk, lipid transfer proteins
Metals	Reaction through direct skin contact	Nickel

**Table 3:** Limitations of diagnostics and treatment of allergic diseases Abbreviations: SPT—Skin Prick Test, CRD—Component-Resolved Diagnostics, IgE—Immunoglobulin E, NSAIDs—Non-Steroidal Anti-Inflammatory Drugs, AIT—Allergen Immunotherapy, IL—Interleukin [20,21,22,23,24,25].

Limitation	Comment
Diagnostics	
Low sensitivity/specificity of skin prick tests	- May lead to false positive (especially in polysensitized patients) and negative results - Possible absence of thermolabile proteins - Diagnostics of allergy caused by IgE-dependent mechanisms
Atypical symptoms	- e.g., Kounis syndrome, acute pancreatitis
Allergy as a mask of primary immunodeficiencies	- e.g., Wiskott–Aldrich syndrome, hyper-IgE syndrome, Netherton syndrome
Variable timing of symptom onset	- Symptoms may be immediate or delayed, complicating diagnosis
IgE tests do not reflect clinical reactivity	- High IgE levels may not correspond to symptoms - Serum IgE half-life ~2–3 days
Lack of access to CRD in many countries	- Limits precision diagnostics for peanut, hazelnut, and other allergens - Specific allergen components may require different dietary management
No biomarkers for non-IgE mediated allergy	- Diagnosis of non-IgE-mediated food allergy relies mainly on elimination diets and challenge tests
Presence of cofactors	- Exercise, NSAIDs, alcohol may trigger or enhance allergic reactions
Hidden allergens	- Unlabeled or unexpected sources in processed foods
Multiple histamine-rich foods	- Overlap with histamine intolerance; difficult to identify single triggers
Oral food challenge	- Available only in highly specialized centers - Requires experienced medical supervision
**Treatment**	
Limited availability of allergen immunotherapy (AIT)	AIT not available or reimbursed in many healthcare systems
Antihistamines	Side effects Often incomplete control of symptoms
Poor adherence to elimination diets	Adherence to elimination diets vary at different developmental phases—increasing independence of adolescents means more risk-taking behaviors.
Lack of curative treatment for food allergy	Only avoidance and emergency treatment (e.g., epinephrine) available
Underuse of biologics in severe allergy	High cost and limited access restrict the use of anti-IgE, anti-IL-4/13 therapies

**Table 4:** Characteristics of selected allergens causing specific symptoms of allergic reactions [28,30,37,39,40,41,42,43,44,45].

Substance/Product	Main Allergen	Protein Class	Characteristics	Cross-Reactivity/Notes
Mustard	Sin a 1	2S-albumin	Very high allergenicity; 0.05 mg causes symptoms	Hidden in meals; possible pancreatitis trigger
Banana	Mus a 1–6	Profilin, chitinase, LTP, thaumatin-like	Triggered with cofactors (e.g., NSAIDs, exercise)	Cross-reacts with birch pollen
Kiwi	Act d 1, d 9, profilin	PR-10, thaumatin-like, profilin	Causes PFAS; cross-reacts with birch	Pollen-food syndrome contributor
Cow’s milk	Bos d 5	Lipocalin (β-lactoglobulin)	Strong allergen, especially in children	Rarely cross-reactive; pancreatitis reported
Fish (tuna, mackerel)	Parvalbumin	Calcium-binding protein	Heat and digestion resistant	Cross-reacts with other fish; FDEIA risk
NSAIDs (e.g., diclofenac)	—	Non-immunologic cofactor	Trigger allergic reactions via COX-1 inhibition	FDEIA, Kounis syndrome cofactor

## Data Availability

No new data were created or analyzed in this study. Data sharing is not applicable to this article.
